# Abundances of Demersal Sharks and Chimaera from 1994-2009 Scientific Surveys in the Central Mediterranean Sea

**DOI:** 10.1371/journal.pone.0074865

**Published:** 2013-09-23

**Authors:** Sergio Ragonese, Sergio Vitale, Mark Dimech, Salvatore Mazzola

**Affiliations:** 1 Institute for Coastal Marine Environment (IAMC), National Research Council (CNR), Mazara del Vallo, Sicily, Italy; 2 Malta Centre for Fisheries Sciences (MCFS), Fort San Lucjan-Marsaxlokk, Birzebbuga, Malta; Aristotle University of Thessaloniki, Greece

## Abstract

Bibliographic and data gathered in scientific bottom trawl surveys carried out off the Southern Coasts of Sicily (Mediterranean Sea), from 1994 to 2009 and between a depth of 10 and 800 m, were analysed in order to prepare a checklist of demersal sharks and chimaera, which are species sensitive to fisheries exploitation. Out of the 27 previously reported demersal shark and chimaera *taxa* in the Mediterranean, only 23 were found in literature and 20 sampled during the surveys in the investigated area. Among the species sampled in the surveys, only 2 ubiquitous ( *Squalus* *blainville* and *Scyliorhinus* *canicula* ) and 3 deep-water ( *Chimaera* *monstrosa* *,* *Centrophorus* *granulosus* and *Galeus* *melastomus* ) species showed a wide geographical distribution with a consistent abundance. Excluding the rare (such as *Oxynotus* *centrina* ) or uncommon shark (e.g. *Squalus* *acanthias* ), the estimated frequencies of occurrence and abundance indexes show a possible risk of local extinction for the almost exclusively (e.g. angelshark, *Squatina* spp.) or preferential (e.g. *Scyliorhinus* *stellaris* ) neritic species.

## Introduction

Chondrichthyes are generally considered among the most sensitive species even to low fishing pressures. In particular, demersal sharks and chimaera share the same life traits of their benthic batoid and pelagic counterparts, including a high position in the trophic food webs, slow growth, delayed sexual maturity, low fecundity and long life spans [1-4]. These animals have always represented a common by-catch of the bottom trawl fleets operating on the fishing grounds located between Southern Sicily and the Northern Coasts of Africa since the 70’s and 80’s. In these decades, bottom trawl activities have increased to cover all the zones off the southern coasts of Sicily, with an ever increasing size, power and efficiency of fishing vessels, which by time moved from the coastal shelf to distant offshore grounds.

It is likely that the overall shift in fishers’ activity and fishing pattern has produced a drastic effect on demersal sharks and chimaera populations and communities for three main reasons.

Firstly, Sicilian fishers believed that the eradication of non-valuable demersal shark in the pristine grounds was a prerequisite to increase the abundance of highly prized shrimps [5]. This belief found some support also in scientific literature since it was thought that sharks were predators of red shrimps and human’s competitors [6-8]. However, more recent studies have shown that shark do not feed on red shrimps [1,9,10].

Secondly, it was not possible to collect information during the development period since only large specimens of few demersal sharks categories (such as *Squalus* spp. or *Mustelus* spp.) were retained on board. As a matter of fact, only few categories of sharks resulted as commercial species according to the past official Italian statistics [11-13], and only recently more attention, both as regulations and data acquisition, have been devoted to selachians [14].

Thirdly, as demonstrated by selectivity studies [10], the narrow mesh size used in the commercial cod-ends (diamond, 20~30 mm side stretched), and the long haul trawling time (up 5-6 hours in slope, 200-800 m bottoms), resulted in almost all the shark specimens being retained in the cod-end and mostly discarded at sea. This practice occurred both in the past [15] and in the present days [16].

The information on demersal sharks and chimaera off the Southern Coasts of Sicily remained scarce even though scouting surveys were implemented in order to explore new potential fishing grounds between the 60’s and the beginning of the 80’s [6,8,15,17,18-20]. The very low average commercial value of sharks in those periods, along with the limited conceptual framework and concern about the modern Ecosystem Approach to Fisheries (EAF), makes historical data on sharks only suitable for qualitative comparisons. For example, Giudicelli [18] reported local abundance of the commercial cartilaginous fish in Sicilian landings in six categories: Angel ( *Squatina* *squatina* ), Ray (all species mixed), Dog fish (large sized *Squalus* *acanthias* *,* *Scyliorhinus* *canicula* and *Mustelus* *mustelus* ), Guitar fish (undetermined, but likely Rhinobatids), “Shark” ( *Hexanchus* *griseus* ), and “Pistin” (a mixture of previous category, plus other bony fish species).

Landing sharks in mixed boxes remained a typical custom of fishers [21], who reported large “bestini” (mix category of large specimens of *Lophius* spp., *Mustelus* spp., *Raja* spp.), small “bestini” (small cartilaginous fish), “agugliata” ( *Etmopterus* *spinax* ), and “rai” (*Raja* spp.).

A regular sampling scheme for the demersal species of the grounds off the Southern Coasts of Sicily started only in the mid 80s with the implementation of the first scientific experimental random depth stratified bottom trawl surveys [22] based on the Sicilian commercial “Mazarese tartana” gear used at that time [23].

Another source of standardized scientific information is obtained from an International program launched in 1994 [24-26] based on a high vertical opening trawl net, the MEDITS program [27,28].

Nowadays, the knowledge about the status of the demersal sharks and chimaera off the Southern Coasts of Sicily remains quite scanty and incomplete. The local faunistic features have been recently reviewed for the Tunisian [29,30], Maltese [31] and all Mediterranean waters [32], whereas only data on distribution, abundance and limited information on specific growth have been published for the Sicilian waters [10,16,24,26,33-37].

The aim of this paper consists in producing a checklist, integrated by occurrence and abundance figures, of the present day and previously reported demersal sharks and chimaera in the investigated area on the base of both historical and scientific survey data.

## Materials and Methods

**Figure 1:**
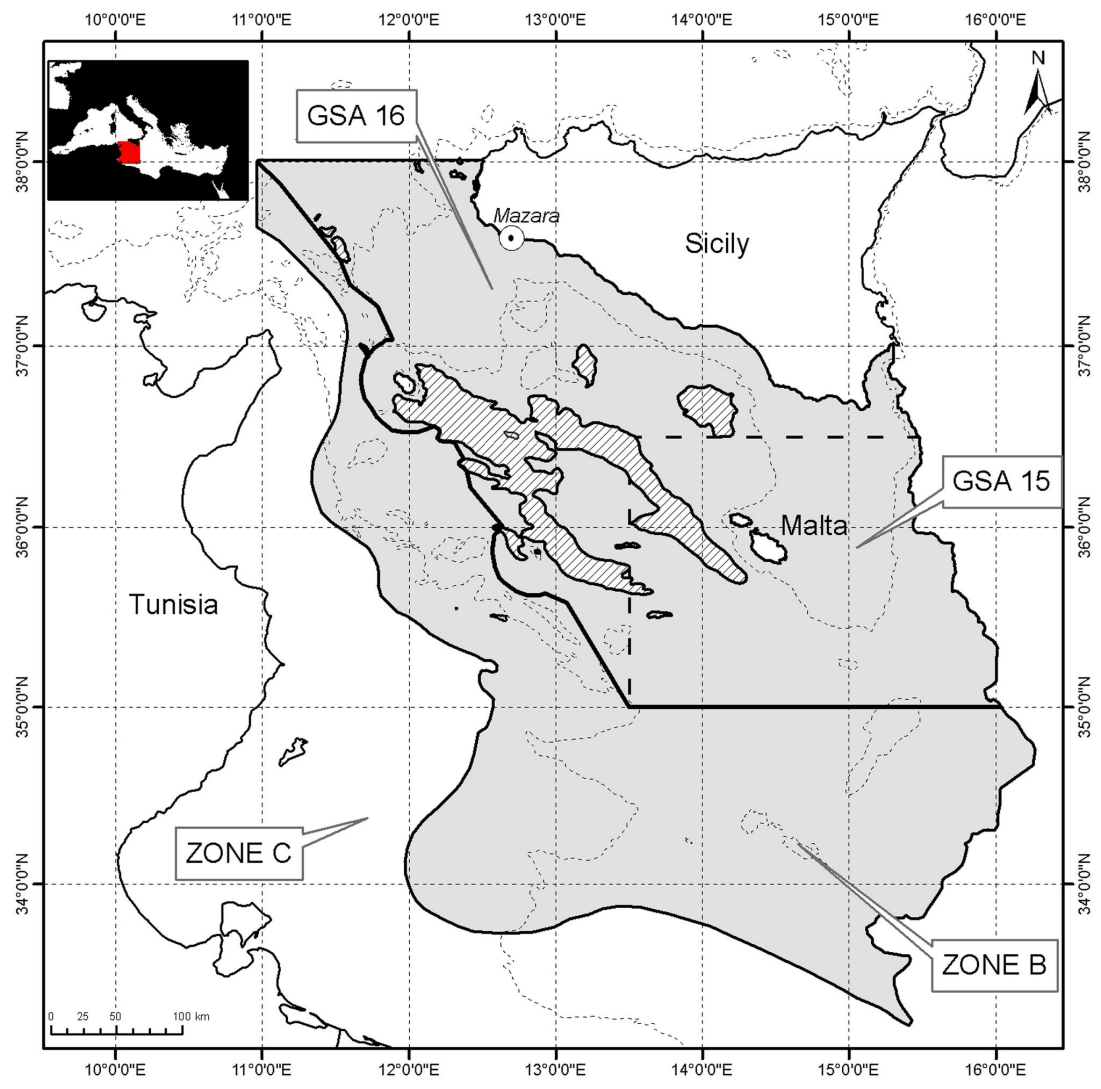
The grounds between the Southern Coasts of Sicily and the Northern Coasts of Africa; the area is over-imposed with the geographical zones considered in the present contribute: South Sicily (GSA 16), Malta Islands (GSA 15) and transitional midline (zone B). The overall extension is 100000 km^2^ and the dotted line denotes the 200 m depth level.

The analysed data refer to a wide area located between the Southern Coasts of Sicily, including the Maltese Islands, and the Northern Coasts of Tunisia and Libya (Figure 1). It includes the continental shelf, slope and other morphologic sub-units such as seamounts (“guyots”) and “banks” [20]. The most distinctive features of these bathyal layers [38] are the presence of huge scattered hard grounds produced by “deep water white corals” (madrepores, mainly *Lopheliapertusa* Linnaeus, 1758, and *Madrepora* *oculata* Linnaeus, 1758 [39]), and the piling up, year after year, of thousands of limestone slabs, which are used as anchors for Fish Aggregating Devices (FADs) in the fishery of dolphin fish ( *Coryphaena* *hippurus* Linnaeus, 1758). These slabs make extended surfaces on the grounds, between 300 m and 800 m, which make the seabed unsuitable for trawling activities, or even untrawlable altogether [40,41].

Bottom trawling is also forbidden (but such as measure is rarely enforced) within 3 nautical miles from the Sicilian coasts and in some specific grounds (such as the “mammellone” off the North Africa). Furthermore, large restrictions within the 25 Nautical mile Maltese Fisheries Management Zone have been established.

With respect to the hydrological pattern of the depth interval explored by the trawl surveys (10–800 m), three main water layers are traditionally recognized [42]: a) the Atlantic (AW; down to 50–100 m or to 150–250 m, depending on locations); b) the Levantine intermediate (LIW; down to 500–700 m), and c) a deep transitional layer (below 600–700 m).

Average Bottom Sea Water Temperature (BSWT in °C), gathered during most of the recent surveys, denoted a seasonal related variation in the coastal waters (10-50 m; 15.8°C ± 0.30 and 17.2°C ± 0.13 in spring and autumn, respectively), slight variation within the 51-100 m interval (15.0°C ± 0.39 and 15.7°C ± 0.29), and homogeneity (13.9-14.1°C) from 201 m down 700-800 m. It is worth noting that the deep BSWT figures are slightly higher than the previous estimations (~12.8°C), likely reflecting the indirect effects of the so-called Transitional Eastern Mediterranean Deep water, a slightly warmer (13.5°C) and much denser body of water, which originates in the Eastern Mediterranean [43].

**Table 1:** Synoptic list of the twenty-seven *taxa* (demersal sharks and chimera) reported in the Mediterranean Sea.

**Class: *CHONDRICHTHYES***	**Scientific name and Authority**	**Remarks: synonymy and abundance perception in the past**	**MRRL**
**Subclass** Holocephali Order CHIMAERIFORMES			
**Family** CHIMAERIDAE	*Chimaera* *monstrosa* Linnaeus, 1758	*Chimaera* *borealis* ; mainly on the slope, juveniles on the shelf; abundant, totally discarded.	NT
	*Hydrolagus* *mirabilis* (Collett, 1904)	*Chimaera* *mirabilis* ; poorly known small *Hydrolagus* specie only recently reported in the Mediterranean Sea.	
**Subclass** Neoselachii Cohort Selachii			
Superorder Squalomorphii			
**Order** HEXANCHIFORMES			
**Family** HEXANCHIDAE	*Heptranchias* *perlo* (Bonnaterre, 1788)	*Heptranchias* *cinereus* ; rare in Malta and Tunisia, common in the other zones; sold at the market.	VU
	*Hexanchus* *griseus* (Bonnaterre, 1788)	*Squalus* *griseus* ; rare in Tunisia, common in the other zones; sliced and sold at the market.	NT
	*Hexanchus* *nakamurai* Teng, 1962	*Hexanchus* *vitulus* ; rare in the Mediterranean and never reported in the investigated area.	DD
**Order** SQUALIFORMES			
**Family** ECHINORHINIDAE	*Echinorhinus* *brucus* (Bonnaterre, 1788)	*Squalus* *brucus* ; reported in Sicily and, as rare occurrence, around the Malta Islands.	DD
**Family** SQUALIDAE	*Squalus* sp	*Taxon* of uncertainilty identity in the past erroneously attributed *Centrophorus* (*uyato*)*.*	
	*Squalus* *acanthias* Linnaeus, 1758	*Squalus* *acanthias* ; very common and abundant mainly on the shelf; sold at the fish market.	EN
	*Squalus* *blainville* (Risso, 1827)	*Squalus* *fernandinus* ; ubiquist, very common and sold at the fish market.	DD
	*Squalus* *megalops* (Macleay, 1881)	Rare in the Mediterranean and in the investigated area	DD
**Family** CENTROPHORIDAE	*Centrophorus* *granulosus* (Bloch and Schneider, 1801)	*Squalus* *granulosus* ; very abundant mainly on slope; sold in the market	VU
**Family** ETMOPTERIDAE	*Etmopterus* *spinax* (Linnaeus, 1758)	*Centrina* *nigra* ; locally abundant mainly on the slope; usually totally discarded.	LC
**Family** SOMNIOSIDAE	*Centroscymnus* *coelolepis* Bocage and Capello, 1864	*Scymnodon* *melas* ; Western Mediterranean and Crete deep water; never reported in the investigated area.	LC
	*Somniosus* *rostratus* (Risso, 1827)	*Somnioususmicrocephalus* ; rare in the Mediterranean; reported generically off the Sicilian coasts.	LC
**Family** OXYNOTIDAE	*Oxynotus* *centrina* (Linnaeus, 1758)	*Centrinasalviani* ; from rare (Tunisia) to common (other zones); totally discarded.	CR
**Family** DALATIIDAE	*Dalatias* *licha* (Bonnaterre, 1788)	*Scymnorhinus* *licha* ; common, mainly on the slope, rarely sold.	DD
**Order** SQUATINIFORMES			
**Family** SQUATINIDAE	*Squatina* *aculeata* (Cuvier 1829)	*Rhina* *aculeata* ; easily confused with other Angelsharks; reported as common in Tunisia and rare around Malta (its occurrence in Malta was not confirmed).	CR
	*Squatina* *oculata* Bonaparte 1840	*Squatina* *fimbriata* ; easily confused with other Angelsharks; reported as common in Tunisia and rare around Malta (its occurrence in Malta was not confirmed).	CR
	*Squatina* *squatina* (Linnaeus, 1758)	*Squatina* *vulgaris* ; very common, frequently recorded; sold in the market.	CR
Superorder Galeomorphii			
**Order** CARCHARHINIFORMES			
**Family** SCYLIORHINIDAE	*Galeus* *atlanticus* (Vaillant, 1888)	Only in the Alboran Sea; never reported in the investigated area.	NT
	*Galeus* *melastomus* Rafinesque, 1810a	*Pristiurus* *atlanticus* ; abundant mainly on slope bottoms; totally discarded.	LC
	*Scyliorhinus* *canicula* (Linnaeus, 1758)	*Scyllium* *canicula* ; abundant mainly at the break shelf; large specimens skinned and sold at the market.	LC
	*Scyliorhinus* *stellaris* (Linnaeus, 1758)	*Scylliumcatulus* ; abundant in Tunisia, common in the other zones, mainly on the shelf and on the westernmost side of the investigated area; discarded by long range fisheries.	NT
**Family** TRIAKIDAE	*Galeorhinus* *galeus* (Linnaeus, 1758)	*Squalus* *galeus* ; not reported off Sicily, rare but diffuse in Tunisia and around the Maltese Islands.	VU
	*Mustelus* *asterias* Cloquet, 1821	*Mustelus* *stellatus* ; from rare to quite common mainly on the shelf (not present in the Gulf of Gabes); sold at the market.	VU
	*Mustelus* *mustelus* (Linnaeus, 1758)	*Squalus* *mustelus* ; abundant mainly on the shelf; sold at the market.	VU
	*Mustelus* *punctulatus* Risso 1827	*Mustelus* *mediterraneus* ; common in Tunisia, rare elsewhere; sold at the market.	DD

Taxonomy and systematic-species follow Ebert and Stehmann [54] integrated by Cavanagh and Gibson [52] and Iglesias [53]. (MRRL, Mediterranean Regional Red List; CR, Critically Endangered; EN, Endangered; VU, Vulnerable; NT, Near Threatened; LC, Least Concern; DD, Data Deficient).

The investigated area has been heterogeneously explored by scientific surveys due to technical, administrative (available funds and contract requirements) and political (International agreements) constraints, resulting in a limited temporal series in some zones, spatial discontinuity and variable sampling intensity in terms of haul number and location.

In recent years, a post stratification was implemented, mainly taking into account the classification adopted by the General Fisheries Commission for the Mediterranean Sea [44]: South Sicily (Geographical Sub-Area, GSA, 16, from 1994 to 2009, herein SS zone), Maltese Islands (GSA 15, from 1994 to 2004, herein MI zone) and a transitional midline area (B zone; from 1997 to 2004, herein BZ). These zones have been subjected to different fishing pressures: traditionally high and only recently decreased in SS, increasing in the last years in BZ, and moderate in MI [21,45,46].

In order to avoid too optimistic figures, due to the likely probable differences in sub-stock structure [47] and the reported heterogeneity in the investigated areas [16], each zone was analysed separately.

Demersal sharks and chimaera were considered in the present study as those reported in the Mediterranean, described in the investigated area, and sampled in scientific surveys. As regards the species sampled in scientific surveys, data gathered since 1994 in the spring-summer Mediterranean International Bottom Trawl Survey, MEDITS [28] and autumn GRUppo Nazionale Demersali surveys, GRUND [48] were used.

Sampling at sea has always been conducted with the same vessel, a commercial stern trawler harboured in Mazara del Vallo, the *Sant' Anna* (32.2 m length overall; powered with a 736-kW engine). Two types of bottom trawl net were employed: the purpose-built GOC73 (spring-summer [28]) and the typical “Mazarese” [23] commercial “tartana di banco” (autumn [22]); the former with a vertical opening of the mouth larger than the latter (2.4-2.9 *vs* 0.6-1.3 m), but both gears mount a 20 mm side diamond stretched mesh in the cod-end.

A depth-stratified sampling design was adopted and daytime hauls, lasting 0.5-1h were performed; overall, 708 and 1603 hauls were realized in the MEDITS and GRUND, respectively. Considering the zones, the number of MEDITS-GRUND hauls were: 492-603, 216-374 and 0-626 in SS, MI and BZ, respectively. The 0 hauls in BZ reflects the non-inclusion of this zone in the MEDITS. Finally, the number of MEDITS surveys were 16 and 11 in SS and MI, respectively, whereas in GRUND were 13 in SS, 11 in MI and 6 in BZ.

For each surveys’ typology, zone and *taxon* the following evaluations were conducted: a) overall map of the spatial occurrence; b) bivariate (mean number vs 50 m interval depth) plot (a and b, available at the Authors); c) frequency of occurrence (percentage, f%); and d) two abundance indexes AI [28], in weight, Biomass Index (BI; kg/km^2^) and number, Density Index (DI; N/km^2^).

Both f% and AI were estimated for the continental shelf (10-200 m), slope (200-800 m) and overall (10-800 m) depth stratum. The figures were reported on the base of the preferential depth *stratum* of the given species as shelf and slope preferential species or having a wide ecological distribution (overall) species. As concerns the slope bottoms definition, the Carpine’s [38] terminology (bathyal, with epi- 200-500 m, and meso- 500-800 m) was preferred because it corresponds to the Sicilian fishers’ habits [8,15,20]. The correlation among MEDITS-GRUND figures (f% and AI) and years were assessed by zone and species by computing the Pearson linear coefficient (r); significance level was set at p=0.05 (degree of freedom Ns- 2, where Ns denotes the number of surveys considered). The grand mean (the mean of the mean) and the corresponding standard deviation of the surveys figures were computed and tabulated for all identified *taxa* by indicating the number of surveys included in the analysis (in case of sporadic occurrence, only the surveys with positive captures were considered). Finally, for eight selected species considered representative of shelf-, slope- and overall depth interval, the spatial distribution of the positive hauls and the yearly DI were represented graphically by survey’s typology and zone. Differences between the means of the DI, BI and f% in the different zones for *C* *. monstrosa* , *S.* blainville*,* *C* *. granulosus* *,* *E. spinax* *,* *D. licha* *,* *G* *melastomus* , *S.* canicula and *M* *. mustelus* were tested using the parametric 2-sample t-test for the MEDITS survey dataset and the one way analysis of variance (ANOVA) for the GRUND dataset. All the data was checked for normality using the Shapiro-Wilk test.

**Table 2:** Percentage frequency of occurrence (f%), mean Density Index N/km^2^ (DI), mean Biomass Index kg/km^2^ (BI) by depth stratum (10-200 m, shelf; 200-800 m, slope; 10-800 m, overall) for all species sampled in SS, South Sicily and MI, Malta Island (spring-summer) investigated zones between 1994-2009 (MEDITS).

**species**	**Rabbitfish**	**Sharpnose sevengill shark**	**Bluntnose sixgill shark**	**Squalus sp** (see text for details)	**Piked dogfish**	**Longnose spurdog**	**Gulper shark**	**Velvet belly**	**Angular roughshark**	**Kitefin shark**	**Sawback angel shark**	**Smoothback angel shark**	**Angelshark**	**Blackmouth catshark**	**Small-spotted catshark**	**Nursehound**	**Tope shark**	**Starry smooth-hound**	**Smooth-hound**	**Blackspotted smoothhound**
**Zone: Maltese island**																				
Ns	11	11	-	-	-	11	11	11	11	11	-	-	-	11	11	2	-	5	8	-
DS	slope	overall	-	-	-	overall	slope	slope	overall	slope	-	-	-	slope	overall	shelf	-	shelf	shelf	-
f%	30.8	10.2	-	-	-	21.2	22.1	55.0	3.9	11.2	-	-	-	80.0	37.5	7.9	-	5.7	14.4	-
sd	14.4	6.5	-	-	-	7.8	14.0	16.2	4.5	10.0	-	-	-	13.4	17.5	3.7	-	5.8	8.3	-
r	0.55	0.61*	-	-	-	-0.17	-0.23	0.48	-0.42	-0.53	-	-	-	0.13	0.58	-	-	-0.11	-0.32	-
DI	11.8	2.3	-	-	-	25.7	5.5	102.6	0.7	1.4	-	-	-	199.7	93.5	1.7	-	1.4	4.4	-
sd	8.9	1.6	-	-	-	24.5	4.1	61.3	0.9	1.5	-	-	-	89.7	78.5	0.8	-	1.5	2.7	-
r	0.52	0.64*	-	-	-	-0.40	-0.19	0.50	-0.26	-0.61	-	-	-	0.35	0.87*	-	-	0.13	-0.13	-
BI	4.8	6.1	-	-	-	9.7	18.7	5.9	1.1	2.0	-	-	-	29.0	9.3	0.8	-	2.3	17.9	-
Sd	4.9	5.0	-	-	-	3.7	12.0	3.5	1.5	3.4	-	-	-	13.6	8.7	1.0	-	2.8	23.9	-
r	0.68*	0.44	-	-	-	-0.19	-0.30	0.66*	-0.27	0.15	-	-	-	0.29	0.84*	-	-	0.54	-0.03	-
**Zone: South of Sicily**																				
Ns	16	15	16	3	2	16	16	16	16	16	-	-	-	16	16	16	-	5	16	6
DS	slope	overall	slope	overall	slope	overall	slope	slope	overall	slope	-	-	-	slope	overall	shelf	-	shelf	shelf	shelf
f%	39.0	1.5	1.0	2.9	2.8	8.7	13.4	48.5	0.8	8.3	-	-	-	74.3	27.0	1.3	-	2.7	17.6	1.8
sd	8.0	1.3	1.7	0.5	1.7	7.2	6.5	10.5	1.3	5.1	-	-	-	10.1	5.9	3.4	-	2.2	7.8	2.3
r	0.58*	0.55*	-0.44	-	-	0.85*	0.56*	0.70*	0.11	0.67*	-	-	-	0.78*	0.79*	-0.29	-	-0.20	0.46	0.18
DI	12.9	0.2	0.1	1.1	0.5	18.4	3.7	51.1	0.1	1.2	-	-	-	195.1	48.6	0.3	-	1.3	13.6	0.8
sd	4.4	0.3	0.2	0.9	0.5	24.1	2.8	27.3	0.2	0.8	-	-	-	154.1	23.5	0.7	-	1.4	10.9	1.0
r	0.25	0.68*	-0.37	-	-	0.85*	0.59*	0.22	-0.06	0.78*	-	-	-	0.85*	0.36	-0.29	-	-0.08	0.51*	0.18
BI	5.0	0.4	1.0	1.0	0.9	8.5	14.9	3.9	0.2	3.0	-	-	-	28.4	7.6	0.4	-	1.0	15.3	0.6
sd	2.0	0.4	2.3	1.0	0.6	10.6	9.1	1.6	0.4	2.1	-	-	-	19.9	3.0	1.3	-	1.0	12.7	0.7
r	0.14	0.33	-0.31	-	-	0.88*	0.51*	0.58*	0.07	0.82*	-	-	-	0.84*	0.34	-0.27	-	0.02	0.63*	0.47

NS, number of surveys; DS, deep stratum; sd, standard deviation; r, Pearson index.

* Significance level at p=0.05 (degree of freedom NS - 2).

## Results

The review of regional literature [8,10,15,17,20,25,26,29-33,37,49,50] and the analysis of the scientific hauls allowed the production of the checklist in table 1.

Among the 27 demersal sharks and chimaera recognized in the Mediterranean Sea [51-55], which belong to five orders (Chimaeriformes, Hexanchiformes, Squaliformes, Squatiniformes and Carcharhiniformes), four species ( *Hydrolagus* *mirabilis* *,* *Hexanchus* *nakamurai* , *Centroscymnus* *coelolepis* and *Galeus* *atlanticus* ) were never reported in the investigated area. Three species ( *Echinorhinus* *brucus* *,* *Squalus* *megalops* and *Somniosus* *rostratus* ) were reported in the investigated area, but never sampled in the scientific surveys. Synthetic comments concerning the 20 *taxa* (19 species and 1 uncertain *taxon*) sampled in the surveys are presented below. The grand means and corresponding standard deviations and r figures are reported in table 2 (MEDITS) and table 3 (GRUND), while the results of the statistical analyses are reported in table 4 (MEDITS) and table 5 (GRUND).

**Table 3:** Percentage frequency of occurrence (f%), mean Density Index N/km^2^ (DI), mean Biomass Index kg/km^2^ (BI) by depth stratum (10-200 m, shelf; 200-800 m, slope; 10-800 m, overall) for all species sampled in SS, South Sicily, MI, Malta Island and BZ, Intermediate Zone (autumn) investigated zones between 1994-2008 (GRUND).

**species**	**Rabbitfish**	**Sharpnose sevengill shark**	**Bluntnose sixgill shark**	** *Squalus* sp** (see text for details)	**Piked dogfish**	**Longnose spurdog**	**Gulper shark**	**Velvet belly**	**Angular roughshark**	**Kitefin shark**	**Sawback angel shark**	**Smoothback angel shark**	**Angelshark**	**Blackmouth catshark**	**Small-spotted catshark**	**Nursehound**	**Tope shark**	**Starry smooth-hound**	**Smooth-hound**	**Blackspotted smoothhound**
**Zone: Maltese island**																				
NS	11	12	-	1	-	9	8	12	12	10	-	2	1	12	12	12	-	9	12	-
DS	slope	overall	-	slope	-	overall	slope	slope	overall	slope	-	shelf	shelf	slope	overall	overall	-	shelf	shelf	-
f%	53.3	16.6	-	4.3	-	32.8	17.9	67.1	4.9	12.0	-	19.1	9.1	84.5	58.0	4.1	-	25.0	23.1	-
Sd	19.6	10.7	-	-	-	7.6	9.1	18.6	5.2	2.9	-	1.3	-	12.0	11.5	5.1	-	19.2	13.5	-
R	-0.03	0.01	-	-	-	-0.38	-0.27	0.04	-0.65*	0.57	-	-	-	-0.07	0.74*	0.13	-	0.56	-0.28	-
DI	15.1	2.2	-	0.3	-	26.4	2.5	114.7	0.4	0.9	-	3.2	1.3	355.0	130.6	0.4	-	3.4	5.4	-
Sd	8.8	1.9	-	0.6	-	27.1	1.6	65.1	0.4	0.2	-	2.6	1.1	155.2	75.6	0.7	-	3.1	4.4	-
R	0.13	-0.11	-	-	-	-0.24	-0.34	0.69*	-0.70*	0.70*	-	-	-	0.47	0.79*	0.26	-	0.76*	0.04	-
BI	5.5	2.7	-	0.22	-	12.2	7.9	6.6	0.4	1.0	-	2.7	0.2	35.0	15.8	0.6	-	6.8	16.7	-
Sd	3.3	1.4	-	0.40	-	6.5	4.8	3.5	0.5	0.9	-	3.5	0.2	14.1	11.8	0.8	-	7.2	25.8	-
R	0.24	0.32	-	-	-	-0.05	-0.33	0.79*	-0.56	0.12	-	-	-	0.37	0.82*	-0.06	-	0.68*	0.16	-
**Zone: South of Sicily**																				
NS	13	13	2	2	2	10	10	13	13	13	-	-	-	13	13	13	1	2	13	1
DS	slope	overall	slope	slope	slope	overall	slope	slope	shelf	slope	-	-	-	slope	overall	overall	slope	shelf	shelf	shelf
f%	43.1	1.6	3.5	2.4	5.4	15.8	13.4	58.0	1.1	10.2	-	-	-	77.1	31.1	1.2	3.6	4.9	26.5	3.7
Sd	7.1	1.9	2.1	0.1	2.9	5.8	6.0	8.1	1.6	4.3	-	-	-	5.0	7.0	1.0	-	2.9	10.7	-
R	-0.60*	-0.07	-		-	0.25	-0.51	-0.67*	0.06	-0.41	-	-	-	0.06	-0.13	-0.48	-	-	-0.55*	-
DI	10.7	0.2	0.25	0.7	0.4	33.5	2.2	37.9	0.1	0.9	-	-	-	234.8	87.4	0.1	0.2	1.8	16.1	0.9
Sd	3.0	0.3	0.03	0.7	0.2	27.2	1.6	11.3	0.1	0.5	-	-	-	92.4	36.5	0.2	0.5	0.3	11.0	1.6
R	-0.48	0.21	-	-	-	0.92*	-0.25	0.21	0.19	-0.25	-	-	-	0.42	-0.50	-0.03	-	-	-0.54	-
BI	3.7	0.3	4.1	1.2	0.4	12.2	6.3	3.3	0.2	2.0	-	-	-	32.4	12.4	0.1	0.03	2.3	17.2	0.5
Sd	1.3	0.4	1.9	1.5	0.3	9.2	5.4	1.1	0.3	1.5	-	-	-	11.8	4.4	0.2	0.06	2.4	12.1	0.9
R	-0.04	0.08	-	-	-	0.94*	-0.14	0.32	-0.06	-0.02	-	-	-	0.39	-0.53	-0.26	-	-	-0.20	-
**Zone: BZ**																				
NS	6	6	6	1	4	4	4	6	6	6	1	1	-	6	6	6	1	6	6	1
DS	slope	overall	slope	slope	slope	overall	slope	slope	overall	slope	shelf	overall	-	slope	overall	overall	slope	overall	shelf	slope
f%	32.5	17.9	1.1	1.7	1.5	43.7	6.9	28.2	1.7	2.1	2.0	1.1	-	42.8	80.6	4.2	0.8	3.7	14.9	1.7
sd	6.8	2.8	1.0	-	1.1	5.4	4.3	6.4	1.7	1.5	-	-	-	11.1	4.1	0.6	-	3.1	8.1	-
r	-0.70	-0.49	-0.18	-	-0.68	0.38	-0.90	-0.85*	0.43	-0.24	-	-	-	-0.93*	0.91*	0.50	-	-0.31	-0.90*	-
DI	9.8	4.1	0.1	0.5	0.1	54.5	1.1	66.2	0.1	0.1	0.5	0.1	-	230.2	498.8	1.0	0.1	0.5	3.1	0.1
sd	4.0	1.2	0.1	1.7	0.1	9.1	0.7	25.9	0.1	0.1	1.1	0.2	-	104.1	64.1	0.5	0.2	0.4	1.7	0.3
r	-0.83*	-0.39	-0.21	-	-0.65	-0.58	-0.84	-0.39	0.40	-0.28	-	-	-	-0.89*	-0.89*	-0.75	-	-0.13	-0.86*	-
BI	3.6	1.7	0.3	0.3	0.2	29.1	3.5	3.5	0.1	0.2	0.4	2.5	-	19.4	40.4	0.4	0.9	0.7	5.6	0.01
sd	1.4	0.5	0.4	1.0	0.3	4.0	2.2	1.2	0.2	0.2	0.9	5.8	-	7.8	5.6	0.2	3.5	0.6	4.3	-
r	-0.40	-0.10	0.50	-	-0.74	0.05	-0.68	-0.34	0.61	-0.36	-	-	-	-0.84*	-0.71	-0.17	-	0.06	-0.89*	-

NS, number of surveys; DS, deep stratum; sd, standard deviation; r, Pearson index.

* Significance level at p=0.05 (degree of freedom NS - 2).

### Rabbitfish - *Chimaera* *monstrosa* Linnaeus, 1758

This Atlanto-Mediterranean deep-water animal prefers cold waters and occurs in all the Mediterranean, except the North Adriatic [56]. Its depth limits range from the outer shelf down to ca. 1600 m [56]. In the Mediterranean, *C* *. monstrosa* is usually caught by offshore trawlers mostly between 500 m and 800 m [24,57] and immediately discarded [20], even though the flesh is edible.

Off the Southern Coasts of Sicily, it resulted quite common in the trawl catches [15], mainly between 400-500 m [16].

**Figure 2:**
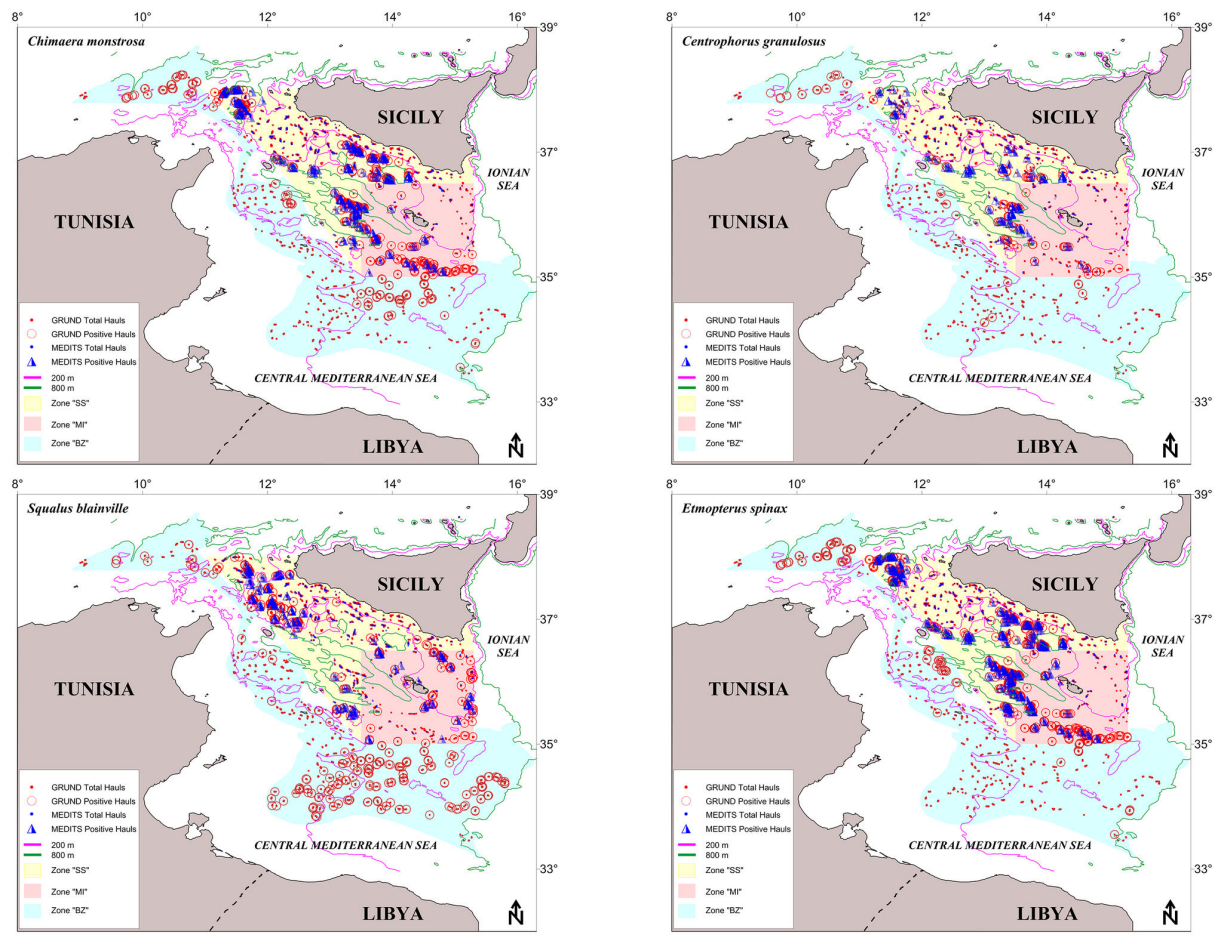
Spatial distribution of *Chimaera* *monstrosa* , *Squalus* *blainville* , *Centrophorus* *granulosus* and *Etmopterus* *spinax* in South Sicily (SS, yellow), Malta Island (MI, light red) and Intermediate Zone (BZ, cyan). Total number of hauls conducted by MEDITS survey from 1994 to 2009 (blue symbols) and GRUND survey from 1994-2008 (red symbols) between the bathymetric of 200 and 800 m are reported.

**Figure 3:**
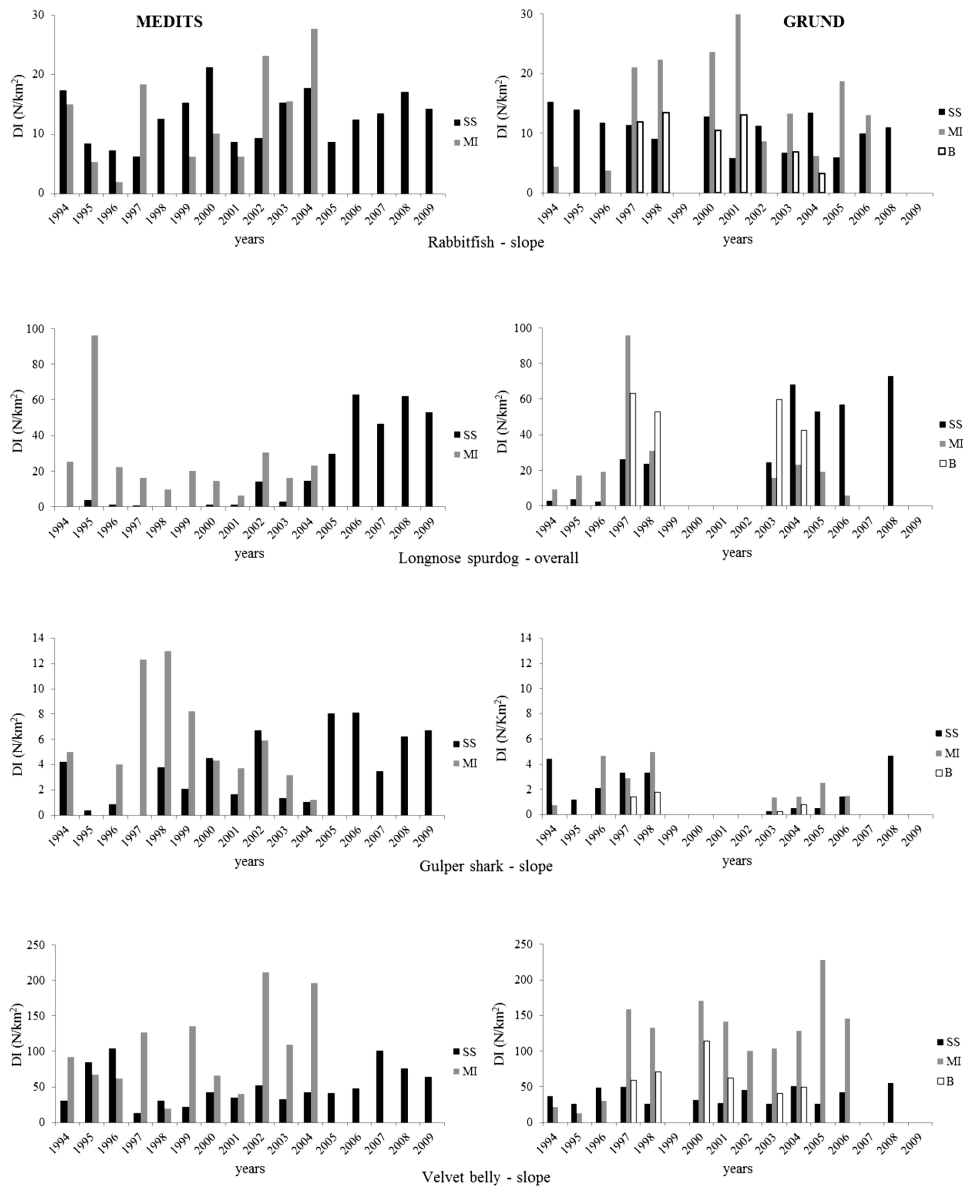
Density Index N/km^2^ (DI) of *Chimaera* *monstrosa* , *Squalus* *blainville* , *Centrophorus* *granulosus* and *Etmopterus* *spinax* by depth stratum (10-200 m, shelf; 200-800 m, slope; 10-800 m, overall) and survey’s typology (MEDITS-GRUND); SS, South Sicily and MI, Malta Island (spring-summer) investigated zones between 1994-2009 (MEDITS), and SS, South Sicily, MI, Malta Island and BZ, Intermediate Zone (autumn) investigated zones between 1994-2008 (GRUND).

Surveys indicated a wide, regular and exclusive occurrence in the bathyal zone (289-799 m) throughout the whole investigated area (Figure 2), hence only this depth interval was considered. The species seems to prefer a narrow depth range; its abundance in number increases to a maximum in the 500-600 m interval, and then decreases in deeper waters. *Chimaera* *monstrosa* was considered as representative of the slope preferential species; the years-DI plot (Figure 3) indicated a fluctuant pattern with higher values in MI in both MEDITS and GRUND. In spring-summer (Table 2) the highest f% (39%), DI (12.9) and BI (5) were recorded in SS; significant positive correlation were found for f% (in SS) and BI (in MI). On the contrary, in autumn (Table 3), the highest f% (53.3), DI (15.1) and BI (5.5) were computed in MI; significant negative correlation resulted for f% (in SS) and DI (in BZ). Furthermore, the statistical analysis showed only a significant difference between the zones for f% and DI in the MEDITS data (Table 4) and in the f% for the GRUND data (Table 5).

**Table 4:** Mean values for Density Index (**DI; N/km** ^2^)**, Biomass Index **(**BI; kg/km** ^2^)** and frequency of occurrence (f%) of the MEDITS dataset with standard deviation, together with the results of T-test analysis between the South Sicily (SS) and Maltese Islands (MI)**.

**Species**		**Mean**		**Standard deviation**		**T-Test statistics**	**P value**
		SS	MI	SS	MI	F	Sig.
Rabbitfish	DI	12.86	11.81	4.40	8.92	8.95	0.01*
	BI	5.01	4.78	2.05	4.86	4.42	0.05
	f%	38.96	30.82	7.97	14.43	5.05	0.03*
Longnose spurdog	DI	18.43	25.66	24.10	24.46	1.15	0.29
	BI	8.51	9.72	10.58	3.72	12.40	0.00*
	f%	8.74	21.22	7.15	7.76	0.00	0.95
Gulper shark	DI	3.71	5.54	2.78	4.13	1.14	0.30
	BI	12.74	17.03	9.47	12.69	0.84	0.37
	f%	13.45	22.08	6.52	14.04	6.37	0.02*
Velvet belly	DI	51.15	102.60	27.29	61.26	7.80	0.01*
	BI	3.87	5.94	1.64	3.48	7.19	0.01*
	F%	48.45	54.99	10.45	16.16	0.92	0.35
Kitefin shark	DI	1.21	1.39	0.81	1.52	2.97	0.10
	BI	2.96	1.99	2.06	3.41	1.08	0.31
	f%	8.26	11.24	5.12	9.99	12.98	0.00*
Blackmouth catshark	DI	195.09	199.68	154.12	89.66	2.16	0.15
	BI	28.36	29.04	19.90	13.60	0.65	0.43
	f%	74.31	79.96	10.13	13.42	0.03	0.85
Small-spotted catshark	DI	48.59	93.53	23.47	78.49	8.29	0.01*
	BI	7.58	9.28	3.05	8.72	7.63	0.01*
	f%	27.00	37.45	5.91	17.55	12.83	0.00*
Smooth-hound	DI	13.64	4.45	10.93	2.72	12.06	0.00*
	BI	15.30	17.91	12.67	23.88	4.06	0.06*
	f%	17.65	14.44	7.78	8.33	0.01	0.92

* Significant difference at 95% confidence intervals.

**Table 5:** Mean values for Density Index (**DI; N/km** ^2^)**, Biomass Index **(**BI; kg/km** ^2^)** and frequency of occurrence (f%) of the GRUND dataset with standard deviation, together with the results of the ANOVA analysis among the South Sicily (SS), Maltese Islands (MI) and the transitional midline area.**

**Species**		**Mean**			**Standard deviation**			**ANOVA**	
		SS	MI	BZ	SS	MI	BZ	F	P value
Rabbitfish	DI	10.66	15.06	9.82	3.04	8.83	3.98	2.15	0.14
	BI	3.66	5.47	3.56	1.26	3.26	1.37	2.36	0.11
	f%	43.11	53.32	32.50	7.13	19.64	6.76	5.01	0.01*
Longnose spurdog	DI	33.48	26.39	54.49	27.23	27.06	9.08	1.72	0.20
	BI	12.19	12.21	29.14	9.20	6.53	3.99	8.24	0.00*
	f%	15.78	32.75	43.65	5.82	7.63	5.38	31.04	0.00*
Gulper shark	DI	2.18	2.52	1.06	1.65	1.58	0.67	1.27	0.30
	BI	6.32	7.88	3.52	5.42	4.82	2.16	1.09	0.36
	f%	13.42	17.91	6.90	5.98	9.13	4.31	3.23	0.06
Velvet belly	DI	37.87	114.70	9.82	11.31	65.07	3.98	16.63	0.00*
	BI	3.33	6.60	3.56	1.07	3.47	1.37	6.80	0.00*
	f%	58.00	67.06	32.50	8.13	18.61	6.76	13.98	0.00*
Kitefin shark	DI	0.94	0.87	0.14	0.54	0.20	0.10	9.47	0.00*
	BI	1.97	0.96	0.19	1.46	0.95	0.19	5.49	0.01*
	f%	10.21	4.35	1.21	11.96	2.89	0.91	16.36	0.00*
Blackmouth catshark	DI	234.79	354.99	230.17	92.45	155.16	104.07	3.62	0.04*
	BI	32.40	34.97	19.39	11.79	14.14	7.82	3.43	0.05
	f%	77.05	84.46	42.76	5.03	12.02	11.09	40.62	0.00*
Small-spotted catshark	DI	87.44	130.65	498.76	36.53	75.62	64.08	105.56	0.00*
	BI	12.43	15.81	40.37	4.42	11.85	5.61	24.78	0.00*
	f%	31.11	57.95	80.61	7.00	11.45	4.12	72.69	0.00*
Smooth-hound	DI	16.06	5.36	3.14	11.04	4.43	1.66	8.34	0.00*
	BI	17.23	16.67	5.57	12.10	25.80	4.26	0.96	0.39
	f%	26.49	23.14	6.08	10.67	13.47	2.99	7.32	0.00*

* Significant difference at 95% confidence intervals.

### Sharpnose sevengill shark - *Heptranchias* *perlo* (Bonnaterre, 1788)

This shark is easily recognizable for the presence of seven gill slits and occurs in the whole Mediterranean showing a wide depth distribution from 0-50 m down to 800-1000 m [24,58,59]. The newborn is ca. 30 cm TL, adulthood reached at 90-100 cm TL, attaints a maximum size of up to 140 cm TL [58].

Off the Southern Coasts of Sicily, the species was reported as a sporadic with low abundance indices in the Gulf of Gabès [32]. It represents an appreciated by-catch in Sicily and Malta.

Surveys indicated heterogeneous spatial distribution with a preference for the central and eastern grounds, i.e., a most common occurrence in MI and BZ. The abundance in number by depth show a parabolic shape with a downward profile (maximum values around 400 m). With the exception of autumn surveys in SS, the species was sampled in both the shelf and epi-bathyal zones, hence the following figures refer to the overall depth interval. In spring-summer, the highest f% (10.2%), DI (2.3) and BI (6.1) were recorded in MI; significant positive correlation were found for f% and DI in both SS and MI. In autumn, the highest f% (17.9), DI (4.1) and BI (1.7) were computed in BZ; no significant correlation was found in any case.

### Bluntnose sixgill shark - *Hexanchus* *griseus* (Bonnaterre, 1788)

A deep-water shark, which has been reported down to 2500m [60] with the ability to approach coastlines during the night up to 30-40m in the Straits of Messina [61]. The species grows up to 600 cm TL [60], and in the mid 80s, large sized animals were commonly found at the fish market in Mazara (Sicily). These specimens were cut, sliced, iced, boxed and exported to the markets of Northern Italy (personal observation). Nowadays, the landings have been reduced in Mazara, but the species is still common in the Maltese fish markets, even if it is worth noting that the Maltese specimens are caught by surface and bottom long-lines [31]. It is sluggish and not aggressive to humans [58], however, it can be harmful to fishers because of the sharp teeth.

Surveys indicated no catch within the Maltese Islands and a few occasional, very scattered (both on spatial and temporal scale), occurrences (MEDITS: f% 1.0 in SS; GRUND: f% 3.5 in SS and 1.1 in BZ) through the bathyal zones (217-706 m) of the SS and BZ. In spring-summer and only in SS, the DI and BI were 0.1 and 1.0, respectively. In autumn, the DI and BI were 0.25 and 4.1 in SS, while in BZ the DI was 0.1 and BI was 0.3; no significant correlation was found in any case.

### *Squalus* sp. Linnaeus, 1758

Considering the systematic uncertainty [54], *Squalus* sp. has been used to refer to specimens classified in both literature and scientific surveys as Little gulper shark, “ *Centrophorus* *uyato* (Rafinesque, 1810)”. This unvalid *taxon* was distinguished from the similar species *C* *. granulosus* mainly according to the shape of the superior teeth and features of the dermal denticles on the sides of the body [15,58]. The “ *C* *. uyato* ” systematic uncertainty is under discussion [53,62], notwithstanding it is still recognised in recent catalogues [63].

As matter of fact, doubts about the validity of the Mediterranean specimens of *C* *. uyato* (for example, considered as the juveniles phase of *C* *. granulosus* ) have been already evidenced in the past by different Authors [15,64]. However, Sicilian fishers do distinguish between the two “forms”, which are often landed and commercialized.

The few specimens referred to *Squalus* sp. were sampled in both spring-summer (1994, 1995 and 2007) and autumn (1997, 2008), with a scattered shelf and bathyal occurrence (72-696 m) in all zones (especially MI).

### Piked dogfish - *Squalus* *acanthias* Linnaeus, 1758

Piked dogfish is a small bottom-dwelling shark with a maximum recorded size of 160 cm TL [58] with a maximum depth of 800 m [37]. According to Serena et al. [37] this species is more abundant in the Adriatic and the Eastern side of the Mediterranean basin. Often confused with *S* *. blainville* , however the body and eye colour, and the presence of white spots on the back are considered the most distinctive features.

Off the Southern Coasts of Sicily, the species was occasionally caught in deep bottoms and it was regularly found in the fish markets; however nowadays it has almost disappeared.

Surveys indicated the non-occurrence of this species in MI and in the shelf of SS and BZ. Occasional localized (SS) and wide although very scattered (BZ) occurrence were recorded on the bathyal zone (355-684 m). Considering the spring-summer, the SS captures were only in 1998 and 2009 at the westernmost side of the area resulting f% (2.8%), DI (0.5) and BI (0.9). In autumn, the scanty catches were recorded in 1994-95 (SS: f% 5.4, DI 0.4 and BI 0.4) and 1997-98, 2003 (BZ: f% 1.5, DI 0.1, BI 0.2).

### Longnose spurdog - *Squalus* *blainville* (Risso, 1827)

A small shark measuring up to 110 cm TL and occurring down to 700 m deep [37]. This species is more abundant in the Western Mediterranean [37] and can be macroscopically distinguished [58,65] from its close relatives (piked dogfish and short-snout spurdog).

Off the Southern Coasts of Sicily, the species is reported as common [20] with specimens up to 90 cm TL on the outer shelf and epi-bathyal (50-600 m) representing a commercialised by-catch, depending on the fishing zone [16]. Recently, Serena et al. [37] have estimated for SS and MI joined a standing stock of 478 t.

Surveys indicated a wide occurrence with a preference for the outer shelf and epi-bathyal zones (50-677 m) of the whole area (Figure 2) which is in agreement with Serena et al. [37]. Considering the overall depth interval (of which the species was considered representative) the years-DI plot (Figure 3) shows higher values in MI and BZ, and a recovering patterns in the last years in SS. In spring-summer, the highest f% (21.2%), DI (25.7) and BI (9.7) were recorded in MI; significant positive correlation were found for f%, DI and BI in SS, confirming the recovery tendency of the last years in this zone. In autumn, the highest f% (43.7), DI (54.5) and BI (29.1) were computed in BZ; significant positive correlation resulted for DI and BI in SS. Furthermore, the statistical analysis showed significant differences between the zones for the BI in the MEDITS data and in the BI and f% for the GRUND data.

### Gulper shark - *Centrophorus* *granulosus* (Bloch and Schneider, 1801)

A common deep water species which grows up to 120 cm TL [25] and lives in a depth range from 50 to 1400 m (Compagno in [4]). Considered abundant off the Southern Coasts of Sicily [20], it has usually a very low commercial value and only large specimens are landed in Sicily and Malta.

Surveys indicated a wide (101-800 m), but almost preferential occurrence through the bathyal (especially below 400 m) of the whole area (Figure 2); some sporadic shelf catches were realized only in the BZ in 1997 and 1998, hence only the bathyal was considered. As representative of the slope species, the years-DI plot (Figure 3) indicated fluctuant pattern, especially in spring-summer, with higher values in MI in both MEDITS and GRUND. In spring-summer, the highest f% (22.1%), DI (5.5) and BI (18.7) were recorded in MI; however, significant positive correlation were found for f%, DI and BI in SS. In autumn, the highest f% (17.9), DI (2.5) and BI (7.9) were computed in MI; significant positive correlation resulted for f% in SS and DI in BZ. The statistical analysis showed only a significant difference between the zones for the f% in the MEDITS data.

### Velvet belly - *Etmopterus* *spinax* (Linnaeus, 1758)

This Atlanto-Mediterranean deep-water shark has a benthic life on the shelf and bathyal zones, from 70 to about 2500 m [66], but mostly below 200 m [24]. It is widely present off the Southern Coasts of Sicily both in the past [20] and at present [16].

Surveys indicated a wide (71-800 m), but almost exclusive occurrence throughout the bathyal zone of the whole investigated area (Figure 2); some sporadic shelf catches were recorded only in the BZ in 2000. The highest abundance in number were recorded in a narrow preferential depth interval (400-600 m), hence only the bathyal was considered. As representative of the slope species, the years-DI plot (Figure 3) indicated higher values in MI in both MEDITS and GRUND. In spring-summer, the highest f% (55%), DI (102.6) and BI (5.9) were recorded in MI; significant positive correlation were found for f% in SS, and BI in SS and MI. In autumn, the highest f% (67.1), DI (114.7) and BI (6.6) were computed in MI; significant positive correlation resulted for both abundance index in MI, on the contrary, negative correlation were found for f% in both SS and BZ. Furthermore, the statistical analysis showed significant difference between the zones for all the survey indicators except the f% for the MEDITS data.

### Angular roughshark - *Oxynotus* *centrina* (Linnaeus, 1758)

This bizarre small dark-coloured shark lives near the shelf edge and epi-bathyal (60 to 660 m [58]) but it is able to reach deeper bottoms (down to 800 m [57]) with a maximum size up to 150 cm TL [25].

Off the Southern Coasts of Sicily, it is caught by trawling or accidentally bottom long-lining, from a few metres until deeper waters, especially on the SE of Pantelleria [8]. It is immediately returned to the sea by fishers from Mazara because they think it will bring bad luck.

Surveys indicated a wide, although scattered, occurrence irregularly distributed between shelf and bathyal hauls (52-741 m), but always with low values, hence the overall depth interval was herein considered. In spring-summer, the highest f% (3.9%), DI (0.7) and BI (1.1) were recorded in MI; no significant correlation was found in any case. In autumn, the highest f% (4.9), DI (0.4) and BI (0.4) were computed in MI; significant negative correlation resulted for f% and DI in MI.

### Kitefin shark - *Dalatias* *licha* (Bonnaterre, 1788)

A benthic to mesopelagic deep-water shark occurring in depths between 90 and 1400 m [67], and grows up to 180 cm TL [25].

The Kitefin shark was commonly recorded (up to 93 cm TL [15]) off the Southern Coasts of Sicily, primarily on the epi-bathyal (300-600 m). It is sometimes sold at the fish markets, but normally it is discarded (especially by Sicilian red shrimp trawlers [20].

**Figure 4:**
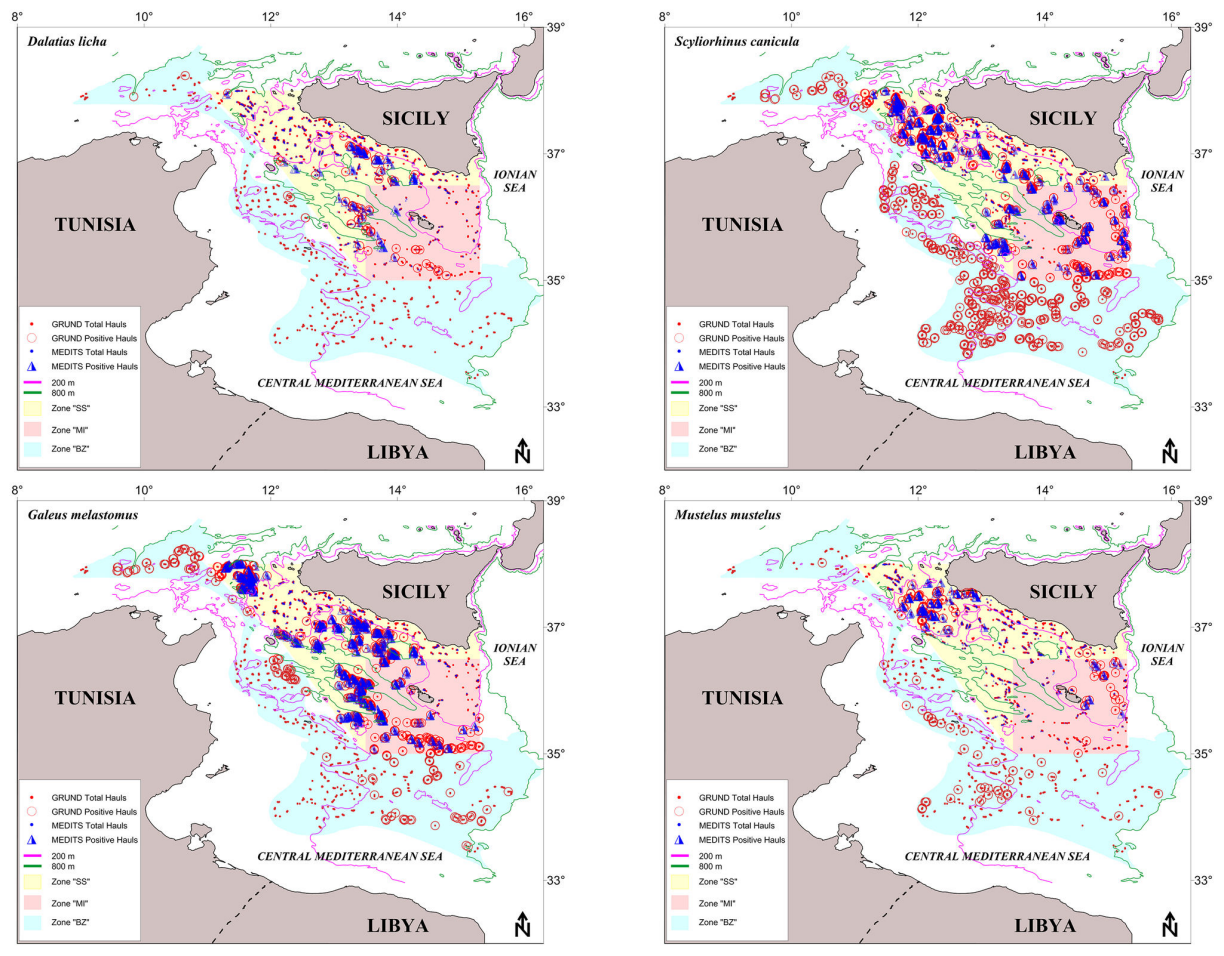
Spatial distribution of *Dalatias* *licha* , *Galeus* *melastomus* , *Scyliorhinus* *canicula* and *Mustelus* *mustelus* in South Sicily (SS, yellow), Malta Island (MI, light red) and Intermediate Zone (BZ, cyan). Total number of hauls conducted by MEDITS survey from 1994 to 2009 (blue symbols) and GRUND survey from 1994-2008 (red symbols) between the bathymetric of 200 and 800 m are reported.

**Figure 5:**
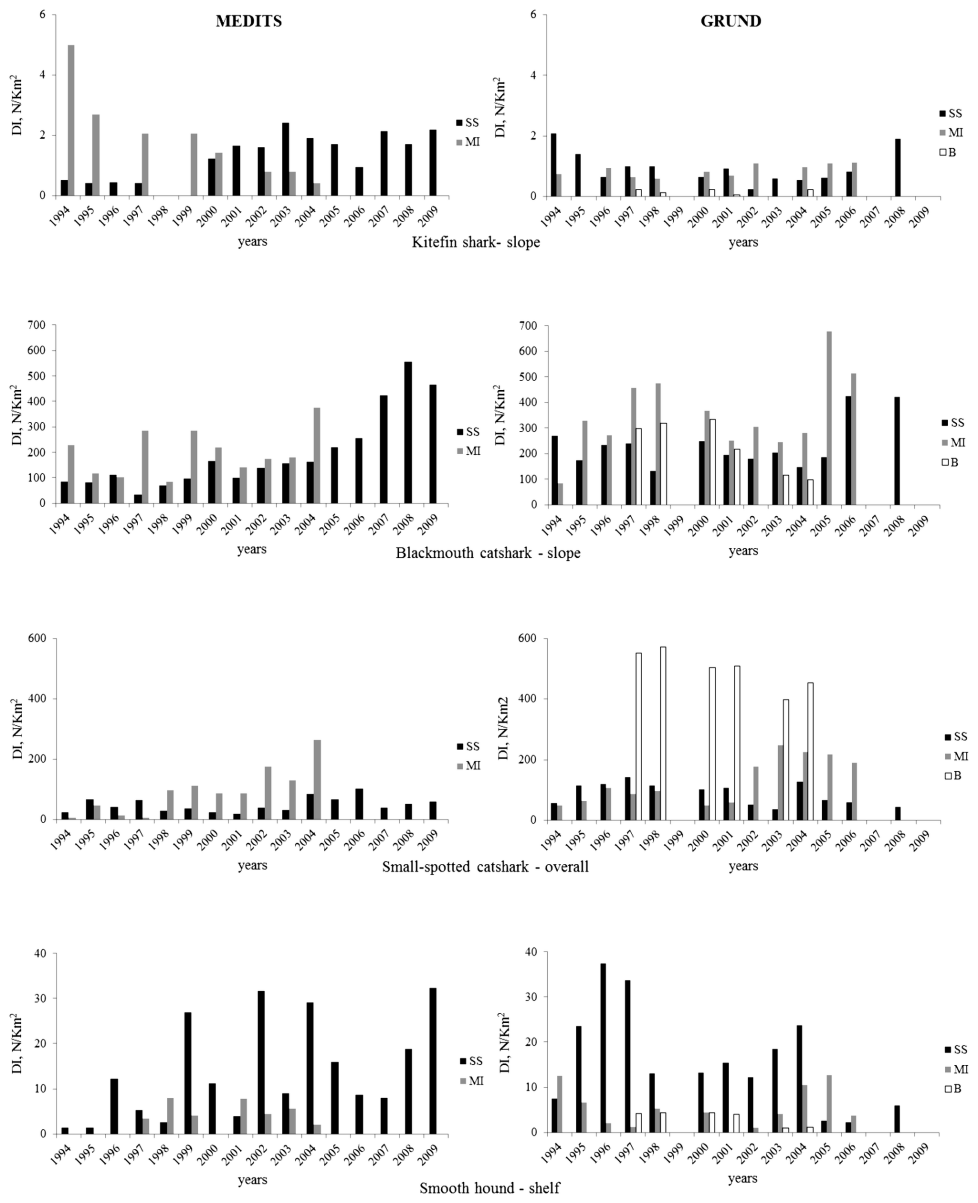
Density Index N/km^2^ (DI) of *Dalatias* *licha* , *Galeus* *melastomus* , *Scyliorhinus* *canicula* and *Mustelus* *mustelus* by depth stratum (10-200 m, shelf; 200-800 m, slope; 10-800 m, overall) and survey’s typology (MEDITS-GRUND) ; SS, South Sicily and MI, Malta Island (spring-summer) investigated zones between 1994-2009 (MEDITS) and SS, South Sicily, MI, Malta Island and BZ, Intermediate Zone (autumn) investigated zones between 1994-2008 (GRUND).

Survey data indicated an exclusive bathyal presence (376-783 m) throughout the whole area (Figure 4), but with a preference for the central and eastern grounds and deeper waters (550-783 m), hence the bathyal zone was considered. As representative for the slope species, the years-DI plot (Figure 5) indicated different patterns: in MEDITS, this species resulted more abundant in MI than SS until 2000, thereafter the opposite trend occurred; in GRUND a slight prevalence in MI is detected. In spring-summer, the highest f% (11.2%) and DI (1.4) were recorded in MI, whereas the highest BI (3.0) was found in SS; significant positive correlation were found for f%, DI and BI in SS. In autumn, the highest f% (17.9) was recorded in MI; the DI (0.9) was the same in SS and MI, whereas BI (2.0) was highest in SS; significant positive correlation resulted for DI in SS. The statistical analysis showed significant difference between the zones for the f% in the MEDITS data and for all the values from the GRUND data.

### Sawback angelshark - *Squatina* *aculeata* (Cuvier 1829)

This angel shark lives at 30 to 500 m depth [58] and grows up to 180-190 cm TL [25,58]; with a preference for the Eastern Basin [68]. Off the Southern Coasts of Sicily, this species was confirmed only in Tunisia [16] and Libya, but always as rare occurrences [29,50].

Surveys indicated the non-occurrence in spring-summer, no matter the zone investigated. Considering the autumn, a sporadic capture was realized in 2000 only in BZ in a shelf haul (86 m) closed to the Gulf of Gabes.

### Smoothback angelshark - *Squatina* *oculata* Bonaparte 1840

An angel shark which grows from 150 cm [25], up to 200 cm TL [69], and prefers depths from 20 to 560 m. The species occurred in Tunisian waters between 50 and 200 m [50] and nowadays it is reported as a rare occurrence [29].

Surveys indicated the non-occurrence in spring-summer, no matter the zone investigated. Sporadic catches in both shelf and slope were recorded in 1997 in BZ and in two shelf hauls in the years 1998 and 2006 in MI (f% 19.1, DI 3.2, BI 2.7).

### Angelshark - *Squatina* *squatina* (Linnaeus, 1758)

This angel shark grows up to 250 cm TL [25], and is common from inshore to at least 150 m depth, but able to reach deeper waters (ca 400 m [69]). Off the Southern Coasts of Sicily, it is the only angel shark commonly reported in the historical faunistic lists [15,19,31], whereas the species was reported between 0-100 m [50]; captures of *S* *. squatina* have been recently reported off Cape Bon [16]. Landings (1980-2008) are characterized by a low production in the Mediterranean and the Black Sea [32].

Surveys confirmed the rarity of this species; only one capture was realized at 128 m in autumn 2005, close to the Maltese Islands.

### Blackmouth catshark - *Galeus* *melastomus* Rafinesque, 1810a

This small sized shark lives in deep waters from 150 down to more than 2000 m [36], even though it can be occasionally (especially juveniles) found over the inner shelf (50-60 m [30,36,70-72]. It reaches 90 cm TL [69,72], with a maximum size in the Mediterranean of 63cm TL [71].

Off the Southern Coasts of Sicily, *G* *. melastomus* is considered common [30] and among the most typical and recurrent species on meso-bathyal grounds [15,20]. Blackmouth catshark is generally considered as having an awful taste and Sicilian fishers discard it immediately [8,16,20].

Surveys indicated a wide (92-800 m), but almost exclusive occurrence throughout the bathyal zones of the whole area (Figure 4). Some occasional and sporadic shelf catches were recorded in SS (autumn 1995 and spring 2006), MI (autumn 2006) and BZ (autumn 1998). The abundance by depth showed a parabolic shape with a downward profile with the maximum abundances between 400 and 600 m, hence the bathyal was considered. As representative of the slope species, the years-DI plot (Figure 5) shows tendencially higher values in MI with signs of increase in SS in the last years. In spring-summer, the highest f% (80%), DI (199.7) and BI (29) were recorded in MI; significant positive correlation were found for f%, DI and BI in SS, confirming the increase tendency detected in the last years. In autumn, the highest f% (84.5), DI (355) and BI (35) were computed in MI. It is worth nothing that significant negative correlation resulted for f%, DI and BI in BZ. The statistical analysis showed only significant difference between the zones for the DI and f% from the GRUND data.

### Small-spotted catshark - *Scyliorhinus* *canicula* (Linnaeus, 1758)

A bottom dweller shark up to 90–100 cm of TL [25,58] and living in gravel, sandy and muddy bottoms down to 800-1000 m, but preferably within the 400–500m depth range [24,73]. Maximum size is usually around 55 cm TL, but for Sicilian waters there is an historical (although not confirmed) record of 100 cm TL, Doderlein in [74].

Off the Southern Coasts of Sicily, it is a very common catch, but only specimens larger than 35cm TL are landed and sold [30].

Surveys indicated a wide and abundant occurrence in both shelf and bathyal (29-794 m) of the whole area (Figure 4), with a preferential depth interval of 100-400 m, hence the overall depth interval was herein considered. As representative of the overall interval species, the years-DI plot (Figure 5) shows differential patterns between MEDITS and GRUND; in the former, higher values were recorded in MI, whereas in GRUND the largest DI were observed in BZ. In spring-summer, the highest f% (37.5%), DI (93.5) and BI (9.3) were recorded in MI; significant positive correlation were found for DI and BI in MI and f% for SS. In autumn, the highest f% (80.6), DI (498.8) and BI (40.4) were computed in BZ; significant positive correlation resulted for f%, DI and BI in MI, while in BZ f% and DI showed a positive and negative correlation, respectively. The statistical analysis showed significant difference between the zones for all the values both from the MEDITS and GRUND datasets.

### Nursehound - *Scyliorhinus* *stellaris* (Linnaeus, 1758)

Its habits are similar to the Small-spotted catshark, but it prefers the rocky zones from 20 to 100 m [25,58], although it is even able to go down to 800 m [24]. Its maximum size ranges from 150 (Mediterranean) to 190 cm TL [69].

Off the Southern Coasts of Sicily, it was captured in some locations (e.g. North Pantelleria [20]) and it is a commonly discarded species [16]; nowadays, it has almost disappeared in many Sicilian fishing grounds and remain common only in Tunisia [30] and Maltese waters [31].

Surveys indicated a very scattered occurrence, with an irregular distribution, on shelf and bathyal (52-667 m). This species showed different occurrence between MEDITS and GRUND. The only capture in MEDITS was realized in SS in the year 2005 (f% 1.7, DI 0.2, BI 0.1). Considering the shelf, in spring-summer the highest f% (7.9%), DI (1.7) and BI (0.8) were recorded in MI; no significant correlation was found in any case. In autumn, the highest values were f% (^≈^4%) in MI and BZ, DI (1.0) in BZ and BI (0.6) in MI; no significant correlation was found in any case.

### Tope shark - *Galeorhinus* *galeus* (Linnaeus, 1758)

A bentho-pelagic ovoviviparous shark growing up to about 200 cm [75] and mainly occurring in the shelf. This very voracious and generalist feeding shark is considered excellent for human consumption [58].

Off the Southern Coasts of Sicily, it was considered as absent in Sicily [49] and rare along the Northern Coasts of Africa [29,50] and Maltese waters [31]; in particular for Malta, the species is reported as “unconfirmed”, even if it may have occurred in the past [31,75]. Nowadays, it is quite common in Tunisia, with very rare occurrences in Sicily and Malta; the species, however, could be more abundant than believed according to small scale fishers in Mazara (personal observation) thus supporting the considerations in [75].

Surveys indicated a very rare, epi-bathyal exclusive (364-570 m), occurrence of this species; few specimens were sampled only in autumn and in the SS (close to the western limit of MI) and BZ (close to the southern limit of MI) in 2002 and 1997, respectively.

### Starry smooth-hound - *Mustelus* *asterias* Cloquet, 1821

A slender shark measuring up to 140 cm TL [58], occurring from a few meters to about 100 m [24], but it can be caught below 300 m [25] and sometimes deeper at 500 m [69].

Off the Southern Coasts of Sicily, this shark is considered rare, while quite frequent in the Tunisian [29] and Maltese waters [31]. Nowadays, it is considered rare in Sicily [16]. This squaliform (and its similar species *M* *. mustelus* ) was landed also in the past [20] since the flesh of the species is well appreciated in Sicily.

Surveys indicated both seasonal and temporal high variability and a wide (70-551 m) although preferential occurrence through the outer shelf (70-200 m) of the whole area. Bathyal captures were never and sporadically (spring-summer 2002; autumn 1997 and 2003) realized in SS and MI, respectively, and more regularly in BZ; hence the shelf was considered in all zones. In spring-summer, the highest f% (5.7%), DI (1.4) and BI (2.3) were recorded in MI; no correlation was found in any case. In autumn, the highest f% (25%), DI (3.4) and BI (6.8) were computed in MI; significant positive correlation resulted for DI and BI in MI.

### Smooth-hound - *Mustelus* *mustelus* (Linnaeus, 1758)

This species measures up to 160 cm TL [69] and lives down to 800 m [24] depth, but showing a preference for shallow sandy-muddy bottoms, especially at 5-50 m depth [25,58].

Off the Southern Coasts of Sicily, it is considered still common [29] as well as in the Gulf of Gabès, where trawl catches consist mainly of immature individuals while longlines catch more mature individual [76].

Surveys indicated a wide (29-557 m) although preferential occurrence through the shelf of the whole area (Figure 4). Epi-bathyal catches were recorded occasionally in SS (spring-summer from 2007 to 2009) and MI (autumn 2005), and more regularly in the BZ, but always with low values; hence the shelf was herein considered. As representative of the shelf species, the years-DI plot (Figure 5) indicated a fluctuating pattern, with higher values in MI in both MEDITS and GRUND. In spring-summer, the highest f% (17.6%), DI (13.6) and BI (15.3) were recorded in SS; significant positive correlation were found for DI and BI in SS. In autumn, the highest f% (26.5), DI (16.1) and BI (17.2) were computed in SS; significant negative correlation resulted for f% in SS and for f%, DI and BI in BZ. The statistical analysis showed significant difference between the zones for the DI and BI from the MEDITS data and for the DI and f% from the GRUND data.

### Blackspotted smoothhound - *Mustelus* *punctulatus* Risso 1826

As the similar species *M* *. mustelus* (with which it is often confused [77]), this shark measures up to 190 cm TL and is reported in the whole Mediterranean [25].

Off the Southern Coasts of Sicily, it is common only in the Gulf of Gabes (Tunisian) and preferably on sea-grass meadows at the inner shelf edge [77].

Surveys indicated the absence in MI and a scattered and occasional occurrence in SS and BZ, between 71 and 303 m. In particular, few specimens were sampled in shelf of SS in 2004, 2008 and 2009 (spring-summer) and 1998 (autumn); only one specimen was sampled in 2004 on the epi-bathyal of BZ. Considering only the spring-summer and the shelf depth interval in SS, the figures were f% (1.85) DI (0.89) BI (0.6); no significant correlation was found in any case.

## Discussion

The present results are in agreement with the scientific literature, which has accumulated in the last two decades: present Mediterranean cartilaginous fish occurrence and abundance appear well below the historical opinion and their catches show a clear decreasing trend [25], with an average overall catch of less than 20,000 t [78].

In particular, this study has shown, on a qualitative base at least, that the present occurrence and abundance of demersal sharks and chimaera in the investigated area are in general less than the previously recorded historical reports. However, the present state seems quite stable or even improving when one considers the correlations for the South Sicily zone (SS). This analysis also supports the fact that the three zones examined have different demersal shark and chimaera features; for example, the highest values were mainly found in the Maltese Islands (MI) and, at least in the first surveys, the Transitional B zone (BZ).

Unfortunately, it is difficult to support in a standardized quantitative manner these findings, due to the fact that there is a lack of statistical commercial data and comparable indexes from the historical period of scientific research. Most of the research was mainly focused on scouting surveys and gear comparisons with heterogeneous objectives, goals and criteria, with different methodologies, which were not standardised.

The positive correlations observed in some cases in spite of the apparent depleted status (for example *M* *. mustelus* MEDITS data for SS) highlight a common problem in interpreting data collected within a relatively short time interval and when the initial or pristine state (i.e., occurrence, abundance and structure of the stock during the development phase of the fishery) is not available cfr. [79]. In these circumstances, stable or even increasing indexes do not necessarily reflect a rebuilding period of the resources, but a temporarily light increase. This is analogous to the right most flat side of the production curve as explained by Hilborn and Sibert [80]. A short time fluctuation in increasing and declining indexes has been, in fact, also reported by Scacco et al. [16].

In the Gulf of Lions, results from scientific trawl surveys [81] indicated that the decline of sharks started in the 60’s on the shelf and extended recently to the bathyal grounds. Only 13 out the 25 species recorded in the years 1957–1960 were still caught in the period 1994-1995. Similar declines were reported by Capapé et al. [82] for the Southern Coasts of France and by Jukić-Peladić et al. [83] in the Adriatic. In the North Tyrrhenian, historical data series [25,84,85] indicated that demersal sharks formed a bigger part of catches in the 50’s than nowadays. At that time there were fisheries targeting specifically demersal sharks such as *S* *. acanthias* and *M* *. mustelus* , which now have almost disappeared. On the contrary, an apparent stable situation (analogous to the present findings) has been reported by Bradai [30], by comparing data for the Tunisian waters (zone C) before and after 1998.

An insight into the trends reported in literature suggests that, excluding the traditional considered rare species (such as *O* *. centrina* ), the response to fishing activities is not always the same [86]. As a matter of fact, sustainability can be theoretically achieved for shark stocks, but some differences in resilience do exist and might be mainly related (as the general case for these animals [87]) to the interaction between different factors [83]. The most relevant factors may include: preferential depth interval, reproduction and feeding pattern (especially scavenger attitudes), surviving capabilities after discarding, and commercial value. In particular, the most neritic (such as *S* *. stellaris* ), ovoviviparous and valuable/appreciated sharks (*Mustelus* spp. and *Squatina* spp.) seem to be the most vulnerable and prone to decline and local extinction. On the contrary, higher resilience is showed by the less valuable, generalist feeders and in large extent discarded (such as *S. canicula*) or fully rejected and deeper dwelling species such as *G. melastomus* and *C. monstrosa*.

The present results are coherent with this interpretation: off the Southern Coasts of Sicily, strictly neritic species are almost locally extinct (*Squatina* spp.) or highly depleted (*S. stellaris*), whereas widely distributed (ubiquist) species are stable (only large *S* *. canicula* are landed) or even increasing at least in SS ( *S* *. blainville* and *M* *. mustelus* ). Furthermore, deep species (for example *G* *. melastomus* ), which habitats go well beyond the usual deepest commercial trawling limit (ca. 750 m), show a stable or even increasing abundance off the Southern Coasts of Sicily, with high resilience to repeated trawling activities [5].

Three explanations can be found for this pattern; first of all, there are evidences that most part of trawled Mediterranean discarded neritic and epi-bathyal sharks were able to survive [88], given the limited temperature variation (due to the homeothermy, especially in winter-spring seasons [42], and the minimum barotrauma suffered (due to the lack of swimming bladder [89].

Secondly, the scavengers or generalist feeders may find at the bottom a large amount of dead bony fish and invertebrates, which are discarded or damaged by trawls which is another food source and a further supply of energy [90].

Thirdly, the recent displacement (mainly out the SS), significant since 2004 of Sicilian large bottom trawlers migrating towards more productive red shrimps fishing grounds [91] have (at least temporarily) mitigated the fishing pressure in the traditional bathyal zones and worsened the situation in the new exploited grounds such as the BZ.

Notwithstanding the previous considerations, it is evident that urgent management measures must be undertaken to protect the demersal sharks and chimaera off the Southern Coasts of Sicily. Traditional natural (white corals) or human induced (FAD grounds and limited access) *refugia*, as well as the permanent 3 nautical miles coastal closure and seasonal fishing ban are not enough. Furthermore, there is the risk that part of the coastal trawlers may migrate to the bathyal zones to replace the decrease in fishing activity of larger trawlers in these grounds. Cod-end selectivity cannot be pursued, at least on the short term, since diamond mesh size of more than 50 mm of opening would be required to get only minimal escapement of the species [92], and Sicilian and Maltese fishers show a strong reluctance in using the 40mm square mesh [93].

Improving trawl design, for example, installing excluding devices or separator grids, protection of nursery and spawning grounds (no take zone) or enlarging present protected areas might represent possible alternatives [94], but such management tools will require a long time for an effective experimentation and implementation or result in poor improvement without a stringent enforcement policy.

An immediate solution could be educational [1], i.e. convincing fishers to return at sea any caught threatened shark, since survival rates for some species can be quite high, such as more than 75% [85,95,96]. This practice would result in minimal economic losses, however with a large conservation benefit in the protection of such a vulnerable group, with a possible solution to stop the decline for these very sensitive animals.
